# The effect of nafamostat mesilate infusion after ERCP for post-ERCP pancreatitis

**DOI:** 10.1186/s12876-022-02345-3

**Published:** 2022-05-31

**Authors:** Joo Seong Kim, Sang Hyub Lee, Namyoung Park, Gunn Huh, Jung Won Chun, Jin Ho Choi, In Rae Cho, Woo Hyun Paik, Ji Kon Ryu, Yong-Tae Kim

**Affiliations:** 1 Division of Gastroenterology, Department of Internal Medicine, Liver Research Institute, Seoul National University Hospital, Seoul National University College of Medicine, 101, Daehak-ro, Jongno-gu, Seoul, 03080 Republic of Korea; 2 Department of Internal Medicine, Dongguk University College of Medicine, Dongguk University Ilsan Hospital, Goyang-si, 10326 Republic of Korea; 3 Department of Internal Medicine, Kyung Hee University Hospital at Gangdong, Seoul, 05278 Republic of Korea; 4 Department of Internal Medicine, Asan Medical Center, University of Ulsan College of Medicine, Seoul, 05505 Republic of Korea; 5 Center for Liver and Pancreatobiliary Cancer, National Cancer Center, Goyang-si, Gyeonggi-do 10408 Republic of Korea

**Keywords:** Nafamostat, Endoscopic retrograde cholangiopancreatography, Post-ERCP pancreatitis

## Abstract

**Background:**

Nafamostat mesilate decreases the incidence of pancreatitis after endoscopic retrograde cholangiopancreatography (ERCP). However, no studies have administered nafamostat mesilate after ERCP. So we investigated if the infusion of nafamostat mesilate after ERCP can affect the post-ERCP pancreatitis (PEP) in high-risk patients.

**Methods:**

In a tertiary hospital, 350 high-risk patients of PEP were reviewed retrospectively. Among them, 201 patients received nafamostat mesilate after ERCP. Patient-related and procedure-related risk factors for PEP were collected. We performed a propensity score matching to adjust for the significant different baseline characteristics. The incidence and severity of PEP were evaluated according to the infusion of nafamostat mesilate. The risk factors of PEP were also analyzed by multivariate logistic regression.

**Results:**

The baseline characteristics were not different after the matching. The PEP rate (17.4% vs. 10.3%, P = 0.141) was insignificant. Among the PEP patients, mild PEP was significantly higher in the nafamostat mesilate group (85.7% vs. 45.5%, P = 0.021). Only one patient in the nafamostat mesilate group developed severe PEP. Although young age (odds ratio [OR] 3.60, 95% CI 1.09–11.85, P = 0.035) was a risk factor, nafamostat mesilate (odds ratio [OR] 0.30, 95% CI 0.09–0.98, P = 0.047) was a protective factor for moderate to severe PEP.

**Conclusions:**

The administration of nafamostat mesilate after ERCP in high-risk patients was not effective in preventing PEP, but may attenuate the severity of PEP.

**Supplementary Information:**

The online version contains supplementary material available at 10.1186/s12876-022-02345-3.

## Background

Endoscopic retrograde cholangiopancreatography (ERCP) is one of the most crucial procedures for diagnosis and treatment of biliary pancreas disease. Nevertheless, we should be careful about the occurrence of adverse effects. Among them, post-ERCP pancreatitis (PEP) is the most common and serious complication of ERCP, occuring in 2–9% of patients [1, 2].

Many studies have been conducted to prevent PEP, and there have been reports that gabexate mesylate, one of the protease inhibitors, can prevent PEP [3, 4]. However, gabexate is inconvenient to use because it has a long infusion time of 12 h and a large dose of the drug is required to prevent expected complications [2, 3, 5]. In one meta-analysis, the number needed to treat (NNT) of gabexate mesylate was 27 [4]. It means that gabexate mesylate should be used in 27 patients to prevent one PEP. Thus, it would be efficient to selectively administer gabexate mesylate to high-risk patients for PEP. The risk of PEP is determined after ERCP due to procedural risk factors. One randomized prospective study showed that gabexate mesylate administered after ERCP was effective in preventing PEP [6].

Several studies have demonstrated that nafamostat mesilate can prevent PEP [7–9]. Previous studies have shown that nafamostat mesilate is more effective than gabexate mesylate because the half-life is 20-times longer and 10–100 times more potent than gabexate [10, 11]. However, nafamostat mesilate was also inefficient to administer to all patients for prophylaxis of PEP. At least 20 mg of nafamostat mesilate should be infused for at least six hours to prevent PEP [7, 9, 12]. The NNT of nafamostat mesilate was up to 31, calculated in a recent randomized controlled trials [7, 12, 13]. Thus, it would be more efficient to selectively use nafamostat mesilate after ERCP in high-risk patients. There was no study in which nafamostat mesilate was administered after ERCP to prevent PEP.

Therefore, the purpose of this study was to investigate the effect of nafamostat mesilate administration after ERCP on the development of PEP in high-risk patients.

## Materials and methods

### Study design and patients

We retrospectively evaluated the prospectively maintained database. We investigated endoscopic sphincterotomy (EST)-naïve patients older than age 19 who underwent ERCP in a tertiary hospital 2016–2018. Among them, high-risk of PEP patients as follow were analyzed; female or young age (< 50), or with a history of acute pancreatitis, a suspected sphincter of Oddi dysfunction, difficult cannulation, precut sphincterotomy, transpapillary balloon dilatation, or multiple pancreatic-duct injections [7]. Exclusion criteria were as follow; history of sphincterotomy, acute pancreatitis before ERCP, pregnant patients, pancreatic head cancer, use of other PEP prophylactic medication (gabexate mesylate, ulistin, etc.) [6, 7].

All procedures were performed by three highly experienced endoscopists with more than 500 cases of ERCP annually. All ERCPs were performed using a standard duodenoscope (TJF-240 or 260 V; Olympus Optical Co., Ltd., Tokyo, Japan). Nafamostat mesilate was administered to the patients at high risk of developing PEP at the physician’s decision after the procedure was completed. Patients of nafamostat mesilate group were infused with 500 mL of 5% dextrose solution with 20 mg of nafamostat mesilate within three hours after ERCP. The infusion continued for six hours [9]. All methods were carried out in accordance with relevant guidelines and regulations. The control group was defined as patients who did not receive any PEP prophylactic medication including nafamostat mesilate.

This study was approved by the institutional review board (IRB) of our university (IRB No. 2016-2038). The need for informed consent was waived by the IRB.

### Data collection and definition

Patient characteristics and ERCP-related data were retrospectively collected. Patients’ characteristics included age, sex, history of acute pancreatitis, sphincter of Oddi dysfunction, indications of ERCP. ERCP-related data included difficult cannulation, pancreatic duct cannulation or injection, precut EST, pancreatic EST, endoscopic papillary balloon dilatation (EPBD), and endoscopic retrograde pancreatic drainage (ERPD).

Difficult cannulation was defined as selective biliary cannulation failing within 10 min regardless of the number of attempts. PEP was defined as serum amylase at least three times higher than normal level at 24 h after ERCP with typical pain. The severity of PEP was classified as mild when hospitalization lasted for two to three days, moderate when four to nine days, and severe when it lasted for more than 10 days or there was pancreatic necrosis, pseudocyst or need for percutaneous drainage or surgery [14]. We defined the hospitalization until the time when symptoms improved and oral feeding started. This is because oral feeding is a crucial factor in determining the length of the hospital stay for pancreatitis [15]. Pancreatic duct manipulation included pancreatic ductal injection and cannulation.

### Study outcome measures

The primary outcome of this study was the PEP rate according to the nafamostat mesilate infusion. Secondarily, we evaluated the severity of PEP according to the nafamostat mesilate infusion and strong risk factors for PEP.

### Statistical analysis

To compare the basic characteristics, Student’s *t*-test and Chi-square test were used for continuous and dichotomous variables, respectively. If any subgroups had less than four subjects, the Fisher’s exact test was used instead of a Chi-square test.

After we had compared the baseline characteristics between the nafamostat mesilate group and the control group, propensity score matching was performed to adjust for significant differences in the baseline characteristics between the two groups. We estimated propensity scores for these patients via multivariate logistic regression analysis and the following two variables were used in the model: difficult cannulation, pancreatic EST. One to one matching between the two groups was performed by the nearest neighbor matching with replacement within 0.05 standard deviations because the nafamostat mesilate group was larger than the control group [16]. As a result of matching with replacement, some control units can be matched to more than one treated unit. The weights was used to reflect the frequency with which each control unit was matched. Thus, we used weighted *t*-test, Chi-square test to compare baseline characteristics after matching. In addition, risk factors for PEP were assessed by weighted logistic regression analysis. Variables with P < 0.1 in univariate analysis were included in a logistic regression model to identify independent risk factors for PEP. P < 0.05 indicated statistical significance. Statistical calculations were performed with SPSS version 19.0 for Windows (SPSS Inc, Chicago, Ill) and R environment ver.3.6.3 (The R Foundation for Statistical Computing, Vienna, Austria). MatchIt package was used for propensity score matching.

## Results

### Baseline characteristics of the study population

**Fig. 1:**
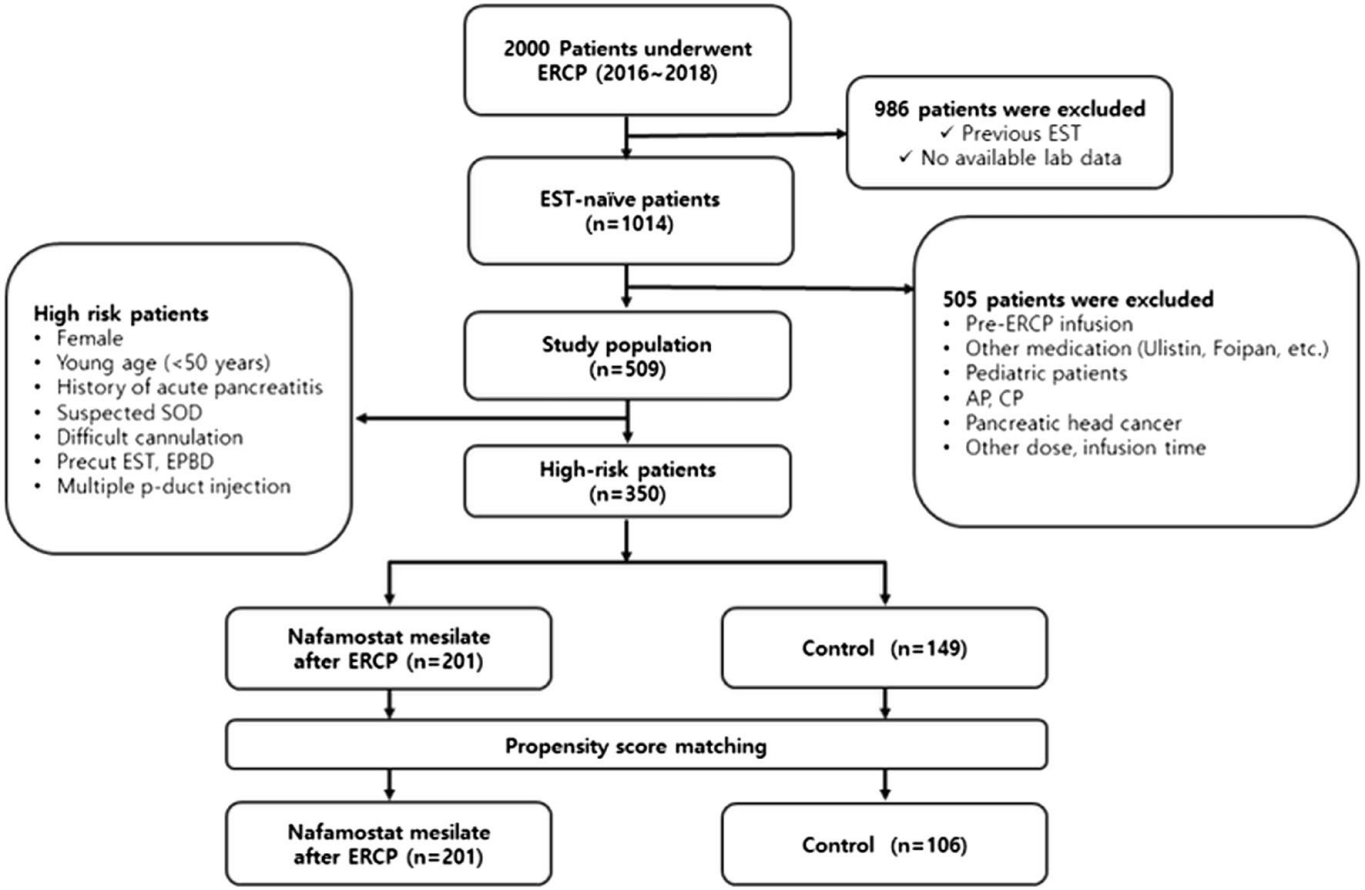
Study flow diagram. *ERCP* endoscopic retrograde cholangiopancreatography, *EST* endoscopic sphincterotomy, *P-duct* pancreatic duct, *SOD* sphincter of Oddi dysfunction, *AP* acute pancreatitis, *CP* chronic pancreatitis

Of the 1014 EST-naive patients, 505 were excluded because of the exclusion criteria thus, a total of 509 patients were reviewed. Among the 509 patients, 350 high-risk patients were analyzed. The nafamostat mesilate group comprised 201 patients and the control group comprised 149 patients (Fig. 1).

The most common indication for ERCP was choledocholithiasis (57.4%, 201 of 350). The rate of difficult cannulation was significantly higher in the nafamostat mesilate group (57.2% vs. 39.6%; P = 0.001). Pancreatic EST (21.9% vs. 13.4%; P = 0.043) was also significantly higher in the nafamostat mesilate group. Other factors did not show significant difference between the two groups (Table 1). After the propensity score matching, the nafamostat mesilate group comprised 201 patients and the control group comprised 106 patients. The difference in the number of patients in each group increased after matching. But it was adjusted by using replacement during matching. There were no significant differences in the baseline characteristics (Table 2). The standardized difference was reduced in all covariates after matching (Additional file 1: Table S1). Moreover, there was no significant difference in the number of risk factors in each group after matching (Additional file 1: Table S2).

**Table 1:** The baseline characteristics in the nafamostat mesilate and the control groups

	Nafmostat mesilate (n = 201)	Control (n = 149)	Total (N = 350)	*P* value
Age, y, median (range)	68 (55–75)	66 (54–74)	66 (55–75)	0.436
Male (%)	74 (36.8)	59 (39.6)	133 (38.0%)	0.596
History of AP (%)	3 (1.5)	0	3 (0.9%)	
SOD (%)	2 (1.0)	0	2 (0.6%)	
Purpose of ERCP (%)				
Choledocholithiasis	120 (59.7)	81 (54.4)	201 (57.4%)	0.318
Malignant biliary stricture	67 (33.3)	56 (37.6)	123 (35.1%)	0.410
Benign biliary stricture	11 (5.5)	8 (5.4)	19 (5.4%)	0.966
Biliary leakage	0	1 (0.7)	1 (0.3%)	
Pancreatic cyst	1 (0.5)	2 (1.3)	3 (0.9%)	0.577
Other indication^a^	2 (1.0)*	1 (0.7)	3 (0.9%)	0.745
Procedures (%)				
Difficult cannulation	115 (57.2)	59 (39.6)	174 (49.7%)	0.001
P-duct manipulation	69 (34.3)	44 (29.5)	113 (32.3%)	0.342
Precut EST	70 (34.8)	42 (28.2)	112 (32.0%)	0.188
Pancreatic EST	44 (21.9)	20 (13.4)	64 (18.3%)	0.043
EPBD	21 (10.4%)	18 (12.1%)	39 (11.1%)	0.631
ERPD	43 (21.4%)	20 (13.4%)	63 (18.0%)	0.055

*AP* acute pancreatitis, *SOD* sphincter of Oddi dysfunction, *ERCP* endoscopic retrograde cholangiopancreatography, *P-duct* pancreatic duct, *EST* endoscopic sphincterotomy, *EPBD* endoscopic papillary balloon dilatation, *ERPD* endoscopic retrograde pancreatic drainage

^a^Intraductal papillary neoplasm of bile duct, Mirrizi’s syndrome

**Table 2:** The baseline characteristics in the nafamostat mesilate and the control groups after matching

	Nafmostat mesilate (n = 201)	Control (n = 106)	Total (N = 307)	*P* value
Age, y, median (range)	68 (55–75)	65 (52–73)	66 (54–75)	0.202
Male (%)	74 (36.8)	47 (44.3)	121 (39.4)	0.186
History of AP (%)	3 (1.5)	0	3 (1.0)	
SOD (%)	2 (1.0)	0	2 (0.7)	
Purpose of ERCP (%)				
Choledocholithiasis	120 (59.7)	56 (52.8)	176 (57.3)	0.401
Malignant biliary stricture	67 (33.3)	42 (39.6)	109 (35.5)	0.260
Benign biliary stricture	11 (5.5)	4 (1.3)	15 (4.9)	0.437
Biliary leakage	0	1 (0.9)	1 (0.3)	
Pancreatic cyst	1 (0.5)	2 (1.9)	3 (1.0)	0.611
Other indication^a^	2 (1.0)	1 (0.9)	3 (1.0)	0.646
Procedures (%)				
Difficult cannulation	115 (57.2)	50 (47.2)	165 (53.7)	1.000
P-duct manipulation	69 (34.3)	36 (34.0)	105 (34.2)	0.229
Precut EST	70 (34.8)	34 (32.1)	104 (33.9)	0.729
Pancreatic EST	44 (21.9)	17 (16.0)	61 (19.9)	1.000
EPBD	21 (10.4%)	14 (13.2)	35 (11.4)	0.893
ERPD	43 (21.4%)	15 (14.2)	58 (18.9)	1.000

*AP* acute pancreatitis, *SOD* sphincter of Oddi dysfunction, *ERCP* endoscopic retrograde cholangiopancreatography, *P-duct* pancreatic duct, *EST* endoscopic sphincterotomy, *EPBD* endoscopic papillary balloon dilatation, *ERPD* endoscopic retrograde pancreatic drainage

^a^Intraductal papillary neoplasm of bile duct, Mirrizi’s syndrome

### Study outcomes

The overall incidence of PEP was 15.0% (46 of 307). There was no significant difference between the nafamostat mesilate group and the control group (17.4% vs. 10.3%, respectively; P = 0.168) (Table 3).

**Table 3:** Incidence and severity of post-ERCP pancreatitis according to the usage of nafamostat mesilate after matching

	Nafmostat mesilate (n = 201)	Control (n = 106)	Total (N = 307)	*P* value
PEP (%)	35 (17.4)	11 (10.3)	46 (15.0)	0.168
Mild (%)	30 (85.7)	5 (45.5)	35 (76.1)	0.021
Moderate to severe (%)	5 (14.3)	6 (54.5)	11 (23.9)	

*PEP* post-ERCP pancreatitis

As a secondary outcome, among the PEP patients, mild PEP was significantly higher in the nafamostat mesilate group (85.7% vs. 45.5%; P = 0.021) (Table 3). There was only one patient with severe pancreatitis in the nafamostat mesilate group. This patient underwent ERCP because of benign biliary stricture, a stricture of anastomosis site after liver transplantation and improved after 12 days of inpatient medical treatment.

In a multivariate analysis, young age (< 50) (OR 3.60 [95% CI 1.09–11.85], P = 0.035) was an independent risk factor for moderate to severe PEP. However, nafamosat mesilate (OR 0.30 [95% CI 0.09–0.98], P = 0.047) was a protective factor (Table 4).

**Table 4:** Univariate and multivariate analysis of the risk factors of moderate to severe post-ERCP pancreatitis

	Moderate to severe PEP			
	Univariate		Multivariate	
	OR (95% CI)	*P*	OR (95% CI)	*P*
Age < 50	3.10 (0.72–11.53)	0.098	3.60 (1.09–11.85)	0.035
Female	3.52 (0.77–16.11)	0.104		
P-duct manipulation	1.11 (0.35–3.51)	0.858		
Precut biliary EST	1.24 (0.39–3.91)	0.717		
Pancreatic EST	0.30 (0.04–2.34)	0.251		
ERPD	1.76 (0.52–5.92)	0.363	1.61 (0.46–5.59)	0.457
Nafamostat mesilate	0.32 (0.10–1.00)	0.051	0.30 (0.09–0.98)	0.047

Multivariate analysis included variables with *P*-value < 0.1 in univariate analysis

*PEP* post-ERCP pancreatitis, *OR* odds ratio, *CI* confidence interval, *P-duct* pancreatic duct, *EST* endoscopic sphincterotomy, *EPBD* endoscopic papillary balloon dilatation, *ERPD* endoscopic retrograde pancreatic drainage

## Discussion

Nafamostat mesilate is effective in preventing PEP. However, it is inefficient to use nafamostat because of the inconvenience in use and high NNT. This study was conducted to use nafamostat mesilate more efficiently. Thus, we investigated the effect of the post-procedural administration of nafamostat mesilate on PEP in high risk patients. As a result, there was no significant difference in the PEP rate, but it had an effect on reducing the severity of PEP.

Many studies have reported that nafamostat mesilate could prevent PEP but was ineffective in high risk patients. Choi et al. [7] reported the results of a well-designed single-center RCT for nafamostat mesilate. They randomized 704 patients and the treatment group was infused with 20 mg of nafamostat mesilate one hour before ERCP and maintained for 24 h. However, there was no significant difference in the PEP rate as a result of the subgroup analysis in high risk patients (12.9% vs 15.2% for control, P = 0.587). In the single center RCT conducted by Yoo et al. [13] in 2011, 50 mg of nafamostat mesilate was administered in the treatment group for six hours. However, there was also no significant difference in the PEP rate of the high-risk patients compared to the control group in the subgroup analysis (6.3% vs 6.5% for control, P = 0.981). In single-center RCT published by Ohuchida et al. [12] in 2015, nafamostat mesilate had no significant effect in high-risk patients (9.7% vs 18.2% for control, P = 0.163). And our study also showed that using nafamostat mesilate after ERCP did not reduce the incidence of PEP in high risk patients.

A meta-analysis conducted by Yu et al. [8] reported that nafamostat mesilate can attenuate the severity of PEP. In this study, the proportion of mild PEP was significantly higher in the nafamostat mesilate group. There were 5 patients in the nafamostat mesilate group and 6 patients in the control group with moderate to severe PEP. In multivariate analysis, nafamostat mesilate had the protective effect for moderate to severe PEP. Hence, the use of nafamostat mesilate in high risk patients after ERCP can attenuate the severity of PEP. It is meaningful because the clinical burden is high when severe PEP occurs.

Although statistically insignificant, the incidence of PEP was higher in the nafamostat mesilate group. It may be because a difficult cannulation and pancreatic EST were conducted more in the nafamostat mesilate group. Thus, NNT could not be calculated from our data. And the overall PEP rate was 15.0%, higher than the previous studies. It was even 17.4% in the nafamostat mesilate group. This may be because this study was conducted on high risk patients of PEP. Additionally, difficult cannulation was very high, approximately by 50%. Other studies reported that the rate of difficult cannulation was approximately 10–36% [7, 12, 13].

In the logistic regression analysis, we did not include difficult cannulation. This is because other procedural risk factors such as precut EST and p-duct manipulation may overlap with difficult cannulation. Although ERPD is a protective factor of PEP, it was included in the multivariate analysis because it is a variable that can impact PEP.

Some patients had a longer hospital stay because they were treated with an underlying disease after improvement of PEP. In these cases, severity may be impacted because severity of PEP depends on the length of the hospital stay. In a previous study, oral feeding was a crucial factor in determining the length of hospital stay for pancreatitis [15]. Thus, we determined the hospitalization duration until symptoms improved and oral feeding started.

This study has limitations. First, selection bias may occur because it is a retrospective study and the administration of nafamostat mesilate was determined after ERCP. However, selection bias would have been reduced via the propensity score matching. Standardized differences of covariates are decreased after matching but still > 0.1. Nevertheless, there is no significant difference in the baseline characteristics after matching, so it can be observed that the matching is balanced. Second, there were confounding factors that could impact the occurrence of PEP such as ERPD and other PEP prophylactic medications. So we excluded patients with other PEP prophylactic medications such as gabexate mesylate and ulistin. And we included ERPD in the multivariate analysis, and there were no significant results other than young age. Additionally, rectal indomethacin was not used in this study because it has not been available in Korea. Hydration was performed when PEP occurred, but it did not reach the amount of proven aggressive hydration [17]. Thus, the impact of PEP by hydration can be considered negligible. Despite these limitations, this study is meaningful as the first study to investigate the impact of nafamostat mesilate infusion after the procedure. And this study differs from other studies in that it only targets high risk patients.

## Conclusions

In conclusion, the use of nafamostat mesilate after ERCP did not prevent PEP, but may have the effect of reducing severity. Thus, nafamostat mesilate may be used after the procedure in high-risk patients expected to develop PEP during the ERCP procedure. Further prospective randomized controlled trial will be needed to support these results.

## Supplementary Information

**Additional file 1: Table S1.** Standardized Differences Before and After the Propensity Score Matching. **Table S2.** The Number of Risk Factors Before and After the Propensity Score Matching.

## Data Availability

The datasets used and/or analyzed during the current study are available from the corresponding author upon reasonable request.

## Abbreviations

ERCP: Endoscopic retrograde cholangiopancreatography
PEP: Post-ERCP pancreatitis
NNT: Number needed to treat
EST: Endoscopic sphincterotomy
EPBD: Endoscopic papillary balloon dilatation
ERPD: Endoscopic retrograde pancreatic drainage
