# Supplementary material for: The effect of nafamostat mesilate infusion after ERCP for post-ERCP pancreatitis
Source: BMC Gastroenterol. 2022 May 31;22:271. doi: 10.1186/s12876-022-02345-3 (PMC9153157; doi:10.1186/s12876-022-02345-3)
Supplement: Supplementary file 1 — Additional file 1: Table S1. Standardized Differences Before and After the Propensity Score Matching. Table S2. The Number of Risk Factors Before and After the Propensity Score Matching. [file 12876_2022_2345_MOESM1_ESM.docx]

**Supplemental Table 1**. **Standardized Differences Before and After the Propensity Score Matching.**

|  | Before matching | | | | After matching | | | |
| --- | --- | --- | --- | --- | --- | --- | --- | --- |
|  | NM | Control | P  value | D | NM | Control | P  value | D |
| Difficult cannulation(%) | 115 (57.2) | 59 (39.6) | 0.001 | 0.358 | 115 (57.2) | 50 (47.2) | 1.000 | 0.202 |
| Pancreatic EST(%) | 44 (21.9) | 20 (13.4) | 0.043 | 0.223 | 44 (21.9) | 17 (16.0) | 1.000 | 0.150 |

NM, nafamostat mesilate; D, standardized difference; EST, endoscopic sphincterotomy.

**Supplemental Table 2**. **The Number of Risk Factors Before and After the Propensity Score Matching.**

|  | Before matching | | | After matching | | |
| --- | --- | --- | --- | --- | --- | --- |
|  | NM | Control | P value | NM | Control | P value |
| Risk factors (mean) | 1.90 ± 0.88 | 1.63 ± 0.83 | 0.001 | 1.90 ± 0.87 | 1.78 ± 0.82 | 0.119 |
| 1 risk factor | 81 (40.3) | 83 (55.7) | 0.004 | 81 (40.3) | 52 (49.0) | 0.141 |
| ≥ 2 risk factors | 120 (59.7) | 66 (44.3) |  | 120 (59.7) | 54 (51.0) |  |

NM, nafamostat mesilate.
